# Supplementary material for: Multi‐dataset identification of innovative feature genes and molecular mechanisms in keratoconus
Source: J Cell Mol Med. 2024 Sep 19;28(18):e70079. doi: 10.1111/jcmm.70079 (PMC11412914; doi:10.1111/jcmm.70079)
Supplement: Supplementary file 2 — Material S2. [file JCMM-28-e70079-s002.zip › 7.GSEA analysis/3.DBNDD1/KEGG_ASCORBATE_AND_ALDARATE_METABOLISM.html]

Details for gene set KEGG\_ASCORBATE\_AND\_ALDARATE\_METABOLISM[GSEA]

|  || Dataset | DBNDD1.group.cls#h\_versus\_l |
| Phenotype | group.cls#h\_versus\_l |
| Upregulated in class | h |
| GeneSet | KEGG\_ASCORBATE\_AND\_ALDARATE\_METABOLISM |
| Enrichment Score (ES) | 0.5230748 |
| Normalized Enrichment Score (NES) | 1.4184058 |
| Nominal p-value | 0.077731095 |
| FDR q-value | 1.0 |
| FWER p-Value | 0.871 |
Table: GSEA Results Summary

  

Fig 1: Enrichment plot: KEGG\_ASCORBATE\_AND\_ALDARATE\_METABOLISM      
 Profile of the Running ES Score & Positions of GeneSet Members on the Rank Ordered List

  

| SYMBOL | TITLE | RANK IN GENE LIST | RANK METRIC SCORE | RUNNING ES | CORE ENRICHMENT || 1 | UGT1A8 | na | 313 | 0.502 | 0.2095 | Yes |
| 2 | UGT2B11 | na | 1393 | 0.345 | 0.3285 | Yes |
| 3 | UGT2B4 | na | 2640 | 0.266 | 0.4083 | Yes |
| 4 | UGT1A6 | na | 2660 | 0.265 | 0.5231 | Yes |
| 5 | UGT2B28 | na | 6027 | 0.150 | 0.4912 | No |
| 6 | ALDH3A2 | na | 12835 | 0.040 | 0.3123 | No |
| 7 | UGT2A3 | na | 13213 | 0.036 | 0.3172 | No |
| 8 | UGT2B10 | na | 17872 | 0.004 | 0.1846 | No |
| 9 | ALDH7A1 | na | 18637 | -0.002 | 0.1634 | No |
| 10 | UGT2B15 | na | 18962 | -0.004 | 0.1559 | No |
| 11 | UGDH | na | 19756 | -0.011 | 0.1378 | No |
| 12 | ALDH1B1 | na | 22410 | -0.036 | 0.0772 | No |
| 13 | ALDH9A1 | na | 22786 | -0.041 | 0.0840 | No |
| 14 | MIOX | na | 23665 | -0.052 | 0.0813 | No |
| 15 | UGT2B7 | na | 24690 | -0.066 | 0.0806 | No |
| 16 | UGT2A1 | na | 28049 | -0.132 | 0.0414 | No |
| 17 | ALDH2 | na | 33235 | -0.347 | 0.0430 | No |
Table: GSEA details [plain text format]

  

Fig 2: KEGG\_ASCORBATE\_AND\_ALDARATE\_METABOLISM      
 Blue-Pink O' Gram in the Space of the Analyzed GeneSet

  

Fig 3: KEGG\_ASCORBATE\_AND\_ALDARATE\_METABOLISM: Random ES distribution      
 Gene set null distribution of ES for **KEGG\_ASCORBATE\_AND\_ALDARATE\_METABOLISM**
